# *Caryospora*-Like Coccidia Infecting Green Turtles (*Chelonia mydas*): An Emerging Disease With Evidence of Interoceanic Dissemination

**DOI:** 10.3389/fvets.2019.00372

**Published:** 2019-10-22

**Authors:** Brian A. Stacy, Phoebe A. Chapman, Heather Stockdale-Walden, Thierry M. Work, Julie Dagenais, Allen M. Foley, Morgan Wideroff, James F. X. Wellehan, April L. Childress, Charles A. Manire, Mya Rodriguez, Trevor T. Zachariah, Lydia Staggs, Bette Zirkelbach, Nina Nahvi, Whitney Crowder, Shane M. Boylan, Shelly Marquardt, Craig Pelton, Terry M. Norton

**Affiliations:** ^1^National Marine Fisheries Service, Office of Protected Resources, University of Florida (Duty Station), Gainesville, FL, United States; ^2^Veterinary-Marine Animal Research Teaching and Investigation Unit, School of Veterinary Science, University of Queensland, Gatton, QLD, Australia; ^3^Department of Comparative, Diagnostic, and Population Medicine, College of Veterinary Medicine University of Florida, Gainesville, FL, United States; ^4^US Geological Survey, National Wildlife Health Center, Honolulu Field Station, Madison, WI, United States; ^5^Florida Fish and Wildlife Conservation Commission, Jacksonville Field Laboratory, Fish and Wildlife Research Institute, Jacksonville, FL, United States; ^6^Loggerhead Marinelife Center, Juno Beach, FL, United States; ^7^Miami Seaquarium, Miami, FL, United States; ^8^Sea Turtle Healing Center, Brevard Zoo, Melbourne, FL, United States; ^9^Veterinary Services, Zoological Operations, SeaWorld Orlando, Orlando, FL, United States; ^10^The Turtle Hospital, Marathon, FL, United States; ^11^Sea Turtle, Inc., South Padre Island, TX, United States; ^12^Gumbo Limbo Nature Center, Sea Turtle Rehabilitation Facility, Boca Raton, FL, United States; ^13^South Carolina Aquarium, Charleston, SC, United States; ^14^Clearwater Marine Aquarium, Clearwater, FL, United States; ^15^Volusia Marine Science Center, Ponce Inlet, FL, United States; ^16^Georgia Sea Turtle Center, Jekyll Island, GA, United States

**Keywords:** *Caryospora*, Eimeriidae, sea turtle, mortality, stranding, pathogen pollution

## Abstract

Protozoa morphologically consistent with *Caryospora* sp. are one of the few pathogens associated with episodic mass mortality events involving free-ranging sea turtles. Parasitism of green turtles (*Chelonia mydas*) by these coccidia and associated mortality was first reported in maricultured turtles in the Caribbean during the 1970s. Years later, epizootics affecting wild green turtles in Australia occurred in 1991 and 2014. The first clinical cases of *Caryospora*-like infections reported elsewhere in free-ranging turtles were from the southeastern US in 2012. Following these initial individual cases in this region, we documented an epizootic and mass mortality of green turtles along the Atlantic coast of southern Florida from November 2014 through April 2015 and continued to detect additional, sporadic cases in the southeastern US in subsequent years. No cases of coccidial disease were recorded in the southeastern US prior to 2012 despite clinical evaluation and necropsy of stranded sea turtles in this region since the 1980s, suggesting that the frequency of clinical coccidiosis has increased here. Moreover, we also recorded the first stranding associated with infection by a *Caryospora*-like organism in Hawai'i in 2018. To further characterize the coccidia, we sequenced part of the 18S ribosomal and mitochondrial cytochrome oxidase I genes of coccidia collected from 62 green turtles found in the southeastern US and from one green turtle found in Hawai'i. We also sequenced the ribosomal internal transcribed spacer regions from selected cases and compared all results with those obtained from *Caryospora*-like coccidia collected from green turtles found in Australia. Eight distinct genotypes were represented in green turtles from the southeastern US. One genotype predominated and was identical to that of coccidia collected from the green turtle found in Hawai'i. We also found a coccidian genotype in green turtles from Florida and Australia with identical 18S and mitochondrial sequences, and only slight inter-regional differences in the internal transcribed spacer 2. We found no evidence of geographical structuring based on phylogenetic analysis. Low genetic variability among the coccidia found in green turtle populations with minimal natural connectivity suggests recent interoceanic dissemination of these parasites, which could pose a risk to sea turtle populations.

## Introduction

The genus *Caryospora* are coccidian parasites in the order Eucoccidiorida, family Eimeriidae. A key feature for morphologic identification is that sporulated oocysts have a single sporocyst with eight sporozoites. The type species is *Caryospora simplex*, which uses snakes in the genus *Vipera* as definitive hosts and can use rodents as intermediate hosts (1). There are at least 81 morphologically described species in the genus (2), but of these, only five named species have available DNA sequence data, and these five do not include the type species, *C. simplex*. Phylogenetic analyses show that *Caryospora* are paraphyletic, with *C. bigemina, Caryospora*-like coccidia from sea turtles, *C. ernsti*, and *C. daceloe/C. neofalconis* representing four distinct clades in the coccidian tree, with *C. daceloe/C. neofalconis* actually clustering in the family Sarcocystidae rather than Eimeriidae (3–6). Although the use of European viper hosts suggests that *C. bigemina* may represent the correct cluster, until reference sequence is available for *C. simplex*, it is not possible to say which one of these clades is actually *Caryospora*.

*Caryospora cheloniae* was first described in the 1970s during the investigation of an epizootic of enterocolitis characterized by dilation, mucosal hyperplasia, and ulceration of the distal small intestine and colon coincident with the presence of numerous coccidia in hatchling and juvenile maricultured green turtles (*Chelonia mydas*) at a facility in the Caribbean (7, 8). Almost 20 years later, a coccidiosis epizootic occurred in wild immature green turtles in Moreton Bay in southeast Queensland, Australia (9). In addition to enterocolitis, extra-intestinal schizonts were found in the brain, thyroid gland, and kidney. Some turtles exhibited abnormal neurological signs attributed to meningoencephalitis from the protozoal infection. Sporadic cases were documented in Australia during subsequent years (10) and another epizootic occurred there in 2014 (4).

Gordon et al. (9) identified the coccidia associated with the first reported Australian epizootic as *C. cheloniae* based on morphology; however, there was no apparent connection between the Australian population of green turtles and the captive-bred green turtles of the original Caribbean report, which were descendants of stock collected from Atlantic nesting assemblages. Genetic characterization of the coccidia from the 2014 Australian epizootic identified two genotypes based on partial sequencing of the ribosomal 18S gene. One genotype was found in coccidia from the gastrointestinal tract, brain, and lung and a second genotype was found in coccidia from the thyroid gland and kidney (4). Phylogenetic analysis suggested that these two genotypes have different relations to other coccidia, highlighting problems with *Caryospora* taxonomy and the putative identification of *C. cheloniae* in sea turtles. As Chapman et al. (4) note, the absence of genetic data from the original Caribbean *C. cheloniae* epizootic and the aforementioned taxonomic uncertainty around recognized *Caryspora* spp. present a formidable barrier to resolving taxonomy and to making a genus or species diagnosis. In acknowledgment of their undefined taxonomic status, hereafter we refer to coccidia found in sea turtles morphologically resembling the original description of *C. cheloniae* as *Caryospora*-like organisms (CLOs).

Coccidia with *Caryospora*-like morphology were not reported in free-ranging sea turtles outside of the Southwest Pacific for years following the initial Australian epizootic despite extensive long-term mortality surveillance efforts in other areas, including the western Atlantic and Hawai'i. To our knowledge, the earliest record of a CLO in another region was the detection of oocysts in a green turtle fecal sample collected from a foraging area near the Marquesas Keys, Florida, US in 2007 (B. Stacy, unpublished data). Four cases of CLO infections were next encountered in green turtles captured in the Gulf of Venezuela from 2011–2013 (11). Around the same time, the first wild green turtles with clinically significant CLO infections were found in the Northwest Atlantic (12, 13). Shortly thereafter, we documented a CLO epizootic concurrent with increased green turtle strandings in southeastern Florida during the fall of 2014 and the winter and spring of 2015. In 2018, we discovered a moribund juvenile green turtle in Hawai'i with severe enteritis associated with a CLO; the first case in this region.

Collectively, this increase in diagnoses within US waters of the Northwest Atlantic and Central Pacific over the last decade suggested a recent change in parasitism or disease occurrence within these areas. These findings prompted a broader collaborative study encompassing locations of known CLO infections in green turtles within the US and Australia to better understand the genetic diversity and distribution of these parasites, and attempt to explain the recent emergence of this disease in the northern hemisphere. Here we describe cases of CLO infection in green turtles, including the epizootic in southeastern Florida, and results of genetic characterization and phylogenetic analysis primarily based on the 18S ribosomal and mitochondrial cytochrome oxidase I (MT-*coI*) genes.

## Materials and Methods

### Sea Turtle Evaluation and Sampling

Participants in the Sea Turtle Stranding and Salvage Network (STSSN) documented stranded sea turtles on the Atlantic and Gulf of Mexico coasts of the US and recorded date, location, species, carapace length [including straight carapace length (SCL) from the nuchal notch to the caudal-most tip], and any apparent external abnormalities as described by Foley et al. (14). Sea turtles were similarly documented by the stranding responders in Hawai'i.

**Figure 1:**
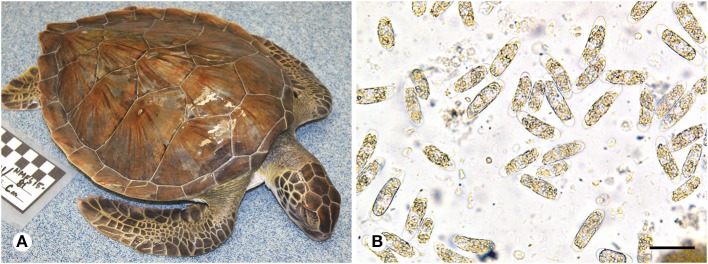
**(A)** Green turtle (*Chelonia mydas*) with coccidiosis found during a 2014/2015 epizootic in southeastern Florida. The frequent presentation of turtles found during this event was absence of injuries, other apparent causes of stranding, or accumulated epibiota. Note the sunken eyes and gaunt neck and shoulder regions due to dehydration or diminished nutritional condition. **(B)** Oocysts morphologically consistent with *Caryospora* sp. (genotype US1) in feces from a green turtle that stranded in Florida. Scale = 40 μm.

**Figure 2:**
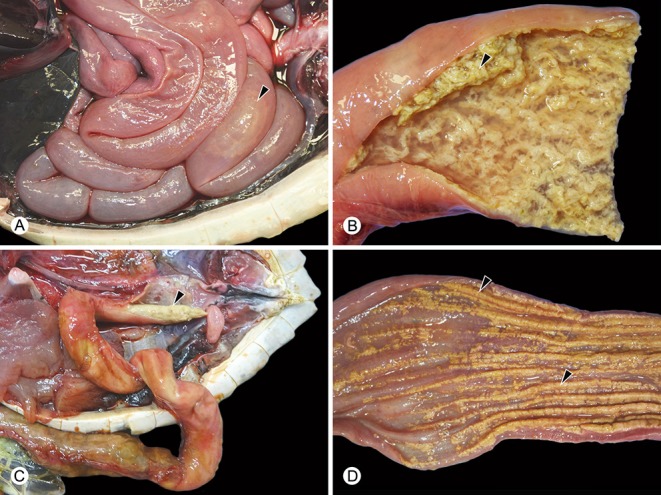
Examples of gross findings in green turtles (*Chelonia mydas*) with enteric *Caryospora*-like coccidiosis. The genotype characterized during this study is provided in parentheses. **(A)** Abnormal watery enteric contents with gas formation (arrowhead) (US1). **(B)** Hyperplasia and sloughing (arrowhead) of the mucosa (US1). **(C)** Colitis (US2) resulting in formation of intraluminal casts of inflammatory exudate (arrowhead). **(D)** Colitis (US1) accompanied by multifocal or diffuse ulceration (arrowheads).

**Figure 3:**
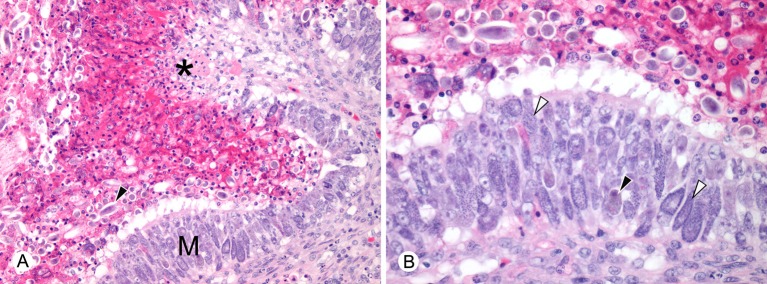
Histopathological lesions in green turtles (*Chelonia mydas*) with enteric *Caryospora*-like coccidiosis (US1 genotype). **(A)** There is hyperplasia of enteric mucosa (M) bordering an area of ulceration (*). Numerous oocysts (arrowhead) are dispersed with heterophilic inflammation in the lumen. Scale bar = 98 μm. **(B)** At higher magnification, large numbers of macrogamonts (black arrowhead) and microgamonts (white arrowheads) are visible within enterocytes. Scale bar = 50 μm. Hematoxylin and eosin.

Diagnosis of CLO infection was based on detection of oocysts in fecal samples or by histopathology consistent with published reports (8, 9) (Figures 1–3). In the southeastern US, live turtles were taken to authorized rehabilitation facilities where diagnostic evaluation included fecal examination for endoparasites by direct smear and flotation. Necropsies were performed on turtles that died during rehabilitation or that were found deceased. Tissues for histopathology were fixed in neutral phosphate buffered formalin, processed into paraffin blocks, sectioned onto glass slides, and stained with hematoxylin and eosin using routine methods. These tissues typically included all major organ systems, including the brain, heart, lungs, liver, gastrointestinal tract, kidneys, spleen, pancreas, thyroid gland, and adrenal glands, but were limited to enteric lesions in some instances due to postmortem condition. For a subset of cases, sections of enteric contents or feces were frozen at −4 or −80°C for genetic study.

For the 2014/15 Florida epizootic, we developed a general case definition that characterized many of the increased strandings during this period. The intent of this case definition was to estimate the total number of stranded animals that may have been affected, but were not examined due to postmortem condition or other logistical reasons, and to examine the role of CLO infection. Our case definition criteria included stranded turtles without any traumatic injury or other external anomaly, including unusual epibiotic accumulation (an indication of chronic illness). In addition, we excluded clearly emaciated turtles but included those with indications of severe dehydration or lesser degrees of weight loss (based on subjective assessment), which are often externally difficult to distinguish and are evidenced by sunken eyes and gaunt neck, shoulder, and prefemoral regions (Figure 1).

### Genetic Characterization and Phylogenetic Analysis

We extracted DNA from enteric contents or feces of green turtles with CLOs using two types of commercial kits (DNeasy® Blood and Tissue Kit, Qiagen, Valencia, California, USA; ZR Fecal DNA Miniprep™ Zymo Research, Irvine, California, USA). The latter was used if failure to amplify DNA was suspected to have resulted from PCR inhibitors in feces. When possible, we concentrated oocysts for DNA extraction using Sheather's sugar flotation and centrifugation (15). In addition, for comparative purposes, we also extracted DNA from CLOs found in feces from loggerhead (*Caretta caretta*) and Kemp's ridley turtles (*Lepidochelys kempii*), as well as an example of *Eimeria carettae* (16) from a loggerhead, the only described eimeriid species other than *C. cheloniae* reported in a sea turtle.

For genetic characterization, we targeted portions of the 18S ribosomal and MT-*coI* genes, and the ribosomal internal transcribed spacer regions (ITS1 and ITS2) (Table 1). For PCR reactions, we used Invitrogen Platinum Taq DNA polymerase (ThermoFisher Scientific, Massachusetts, USA) and the following parameters: denaturation at 94°C for 5 min, then 40 cycles of denaturation at 94°C (45 s), annealing temperature set at 5°C below primer melting temperatures (30 s), and extension at 72°C (60 s). The final extension step was at 72°C for 10 min. Amplicons of the expected size were submitted for Sanger sequencing in both directions (Genewiz, South Plainfield, New Jersey, USA). Sequence of the same genes was obtained from samples from a stranded turtle in Hawai'i and previously reported cases from Australia (4) using separate laboratories at those locations and similar methods.

**Table 1:** Oligonucleotide primers used to amply the partial 18S ribosomal gene, mitochondrial cytochrome oxidase I gene (MT-*coI*), and ribosomal internal transcribed spacer regions (ITS1 and 2).

**Gene**	**Forward**	**F sequence**	**Reverse**	**R sequence**
18S	Cary.18SF	5′-AGTTTCGAGGTAGTGACGAGA-3′	Cary.18S.R2	5′-CCTATCTCTAGTCGGCATAGTTT-3′
	Cary.18S.F2	5′-ACGAACTACTGCGAAAGCAT-3′	Cary.18SR	5′-CATCACAGACCTGTTATTGCCT-3′
	STCoccF	5′-CAAGAACGACAGTAGGGGGT-3′	STCoccR	5′- AGTGATGCGGAAACCAAAGT-3′
ITS1	Coc.NEW.18S.F	5′-CGGAAGGATCATTCACACGT-3′	Coc.ITS2.R	5′-GATGGTTCACTGAAATCTGCAA-3′
	Coc.NEW.18S.F	5′-CGGAAGGATCATTCACACGT-3′	Cary.5.8s.R	5′-CACTGAAATCTGCAATTCACAAT-3′
ITS2	Coc.5.8s.F	5′-ATGAAGRRCGCAGCGAA-3′	Coc.28s.R	5′-TCCTCCRCTTARTAATATGCTTAA-3′
MT-*coI*	Cary.COI.F	5′-ATTTAATTGTAATTGGATTGGCT-3′	Cary.COI.R	5′-CTGTAGGGATAGCAATCATCAT-3′
	Cary.COI.F.2	5′-GTCTATTCACTTGGGCTATTGTATT-3′	Cary.COI.R	5′-CTGTAGGGATAGCAATCATCAT-3′

Chromatographs were evaluated using Finch TV Version 14.0 (Geospiza, Inc., Seattle, Washington, USA). We used Clustal Omega to perform nucleotide sequence alignments and pairwise comparisons (17). Based on previous studies of inter- and intraspecific variation in the 18S and MT-*coI* genes of other apicomplexans (18, 19), unique genotypes were given numeric designations based on MT-*coI* sequence. If any variability was found in the 18S gene from samples with like MT-*coI* sequences, the organism was considered a variant and given an alphanumeric identifier (e.g., 9A, 9B). Genotypes were confirmed by sequences derived from multiple samples. If a genotype was represented by a single example, PCR and sequencing were repeated for confirmation. All chromatographs were reviewed for any nucleotide ambiguity as evidence of potential mixed infections. We required unambiguous 18S and MT-*coI* sequences to designate genotypes to help avoid mismatching of genetic data between types, which is an inherent risk of working with fecal samples that may include different coccidia.

We used the 18S for the phylogenetic analysis based on the additional variation observed in this gene as compared with the MT-*coI*, and the greater availability of 18S sequences of other coccidia in public databases. Five hundred and thirty-four positions were available for analysis after alignment. Smart Model Selection (20) was used to select TN93 as the most appropriate model, with a proportion of invariant sites and gamma distribution. Bayesian inference (BI) analysis was conducted using Mr.Bayes 3.2.6 (21). Three million generations were run, with samples taken every 300 generations. The first 10% of trees were discarded as burn-in, with convergence confirmed using Tracer 1.6 (22). Maximum likelihood (ML) analyses were performed using PhyML 3.0 (23) using the same model and 1,000 bootstrap replicates.

## Results

### Description of 2014–2015 Epizootic and Other Reported US Cases

**Figure 4:**
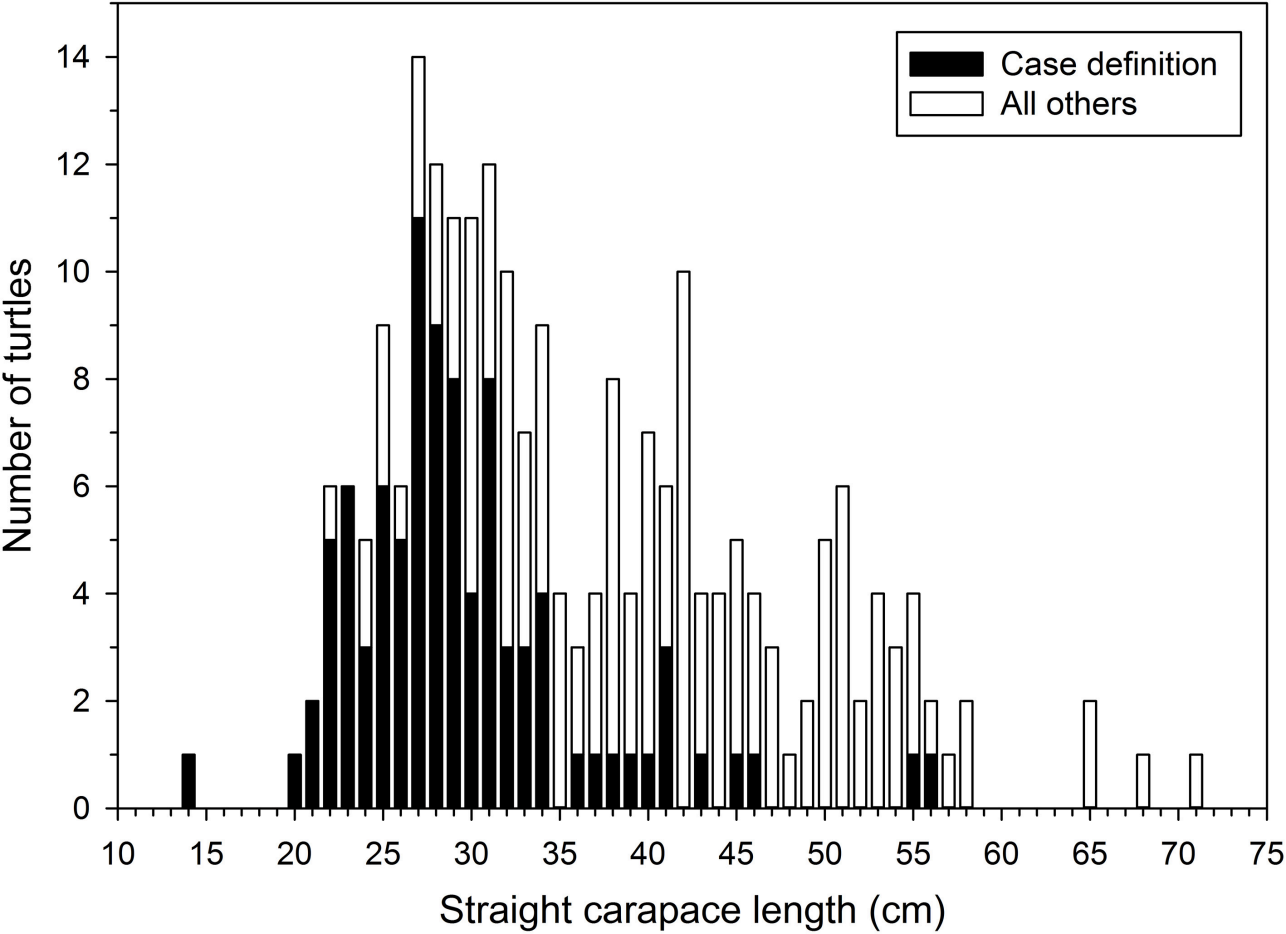
Straight carapace lengths (SCL, nuchal notch to pygal tip) of stranded green turtles (*Chelonia mydas*) found in southeastern Florida during November 2014 through April 2015. The median SCL of turtles matching our case definition for suspected clinically significant *Caryspora*-like organism infections was less than that of all other green turtles that stranded during this same period and area.

In November 2014, rehabilitation facilities began reporting stranded green turtles with coccidiosis along the southern Atlantic coast of Florida (Brevard County through Monroe County). Frequent reporting of these cases continued over the next several months, peaked in April 2015, and tapered off quickly thereafter. This epizootic coincided with a 36.1% increase in stranded green turtles (*n* = 366) relative to the previous 5-year average (*n* = 269) for the same area and time. Ninety-eight of these strandings met our case definition; 60 were found alive, 38 were deceased. The median SCL of these turtles was 27.9 cm (SE = 0.73, range = 13.2–55.5). This was less than that of other stranded green turtles found in these counties during the same time (39.9 cm, SE = 0.88, range = 21.9–71.0; Mann-Whitney U Test, *P* < 0.001) (Figure 4). Fifty of the turtles that met the case definition were screened for coccidia by fecal examination (*n* = 13), histopathology (*n* = 33), or both methods (*n* = 4); 45 were positive for CLOs. Nine of these turtles were found dead, 25 were found alive and later died, and 11 survived and were released following treatment at sea turtle rehabilitation facilities. Of the five turtles meeting the case definition in which CLOs were not detected, two had severe bacterial infections. A cause of stranding was not identified for the other three turtles.

During the epizootic, an additional 13 cases of CLO infection were detected in stranded green turtles that did not meet our case definition, including three turtles found in nearby counties of Volusia and St. Johns. These turtles were either emaciated and had abundant epibiota, or had other apparent causes of stranding, such as advanced fibropapillomatosis, vessel strike injuries, or shark bite wounds. These cases suggest that additional turtles that did not meet our case definition likely had clinically significant CLO infection; however, the numbers of turtles with these other issues that were found during the epizootic were not unusually high (i.e., did not account for the unusually high numbers of strandings during this time).

Turtles diagnosed with CLO infection by histopathology (*n* = 31) had enterocolic lesions like those previously described (8, 9). Grossly, the enterocolic contents were often watery and straw-colored with various degrees of gas formation (Figures 2A,B). Mucosal lesions ranged from hyperplasia to ulceration and were most consistently severe in the distal half of the small intestine, which was filled with fluid and sloughed mucosa. Developing stages of coccidia were numerous within enterocytes and were observed by histology in all areas of the intestine. Myriad oocysts were dispersed among sloughed cells and in the exudate within ulcerated areas (Figure 3). Histopathological examination included brain and other major organs in all 31 cases; no extra-intestinal coccidia were observed.

We concluded that CLO infections were a significant contributing cause of the elevated numbers of strandings during the epizootic based on the frequency of infection among examined stranded turtles (45/50 turtles) and the severity of enterocolic lesions in necropsied individuals. As will be reported elsewhere, some stranded turtles, including those with coccidiosis, also had concentrations of domoic acid or saxitoxins in organs and digestive contents exceeding 200 ng/g, which is above concentrations previously interpreted as asymptomatic exposure in marine mammals [e.g., (24, 25)]; thus, other factors in addition to coccidiosis may have played a causative role in the strandings during this period. Of particular relevance to comparison with prior CLO outbreaks, 24 of 35 live stranded turtles with coccidiosis exhibited lethargy, weakness, and abnormal neurological signs including loss of coordination and abnormal head movements, often in conjunction with severe hypoglycemia (<2 mg/dl). However, we found no histopathological evidence of neurological protozoal infection. Considerations for causes of the neurological abnormalities included hypoglycemia associated with protozoal enterocolitis, as well as biotoxicosis.

Following the 2014/15 coccidiosis epizootic, necropsies conducted by resource agencies and voluntary reporting of coccidial infections diagnosed at rehabilitation facilities throughout the southeastern US (Virginia to Texas) yielded 49 cases of CLO infection in green turtles through December 2018. Additional unreported cases were likely. The stranding locations of most of the infected turtles were distributed along the Atlantic coast of Florida, and included Georgia (*n* = 1) and South Carolina (*n* = 2). Some cases were reported in the Gulf of Mexico, including one case in Florida (Pinellas County) and four from Texas (Cameron County). In contrast to the relatively frequent diagnosis of fatal enterocolitis during the epizootic, most of the subsequent cases were live turtles with infections of indeterminate clinical significance due to multiple concurrent health problems or without any apparent clinical signs. Veterinarians specifically attributed clinical signs (including lethargy, dehydration, and weight loss) to coccidiosis in only 3 of 46 live turtles diagnosed with CLO infection based on fecal examination. To our knowledge, only three green turtles in which coccidiosis was identified as the cause of death have been recorded in the southeastern US since the epizootic (among 226 necropsies conducted in a manner where fatal coccidiosis would have been confidently detected). In addition, a stranded green turtle that died from severe enteric coccidiosis was found on Oahu, Hawai'i on March 8, 2018 representing the first case documented in the Central Pacific region.

In total, a review of available data yielded 113 confirmed cases of CLO infections in green turtles in US waters, including 5 cases prior to the 2014/15 epizootic, 58 during the epizootic, and 49 from subsequent years, as well as the single case from Hawai'i. We were able to genetically characterize the coccidia in 62 of these cases. In addition, we sequenced 18S and MT-*coI* genes from CLOs detected in five loggerhead turtles and two Kemp's ridleys, as well as coccidia from a loggerhead diagnosed as *E. carettae* based on oocyst morphology. As the general parasitological, clinical, and pathological findings associated with CLOs have been previously described, the remaining results focus on those cases in which genetic characterization of the coccidia was possible.

### Genetic Characterization

#### Genotypic Variation

We sequenced a homologous 525 bp partial segment of the 18S gene and 344 bp of the MT-*coI* gene from all genotypes. We were able to sequence larger 810 bp sequences of the 18S gene from some cases and included this extra sequence data in our comparisons and submissions to GenBank; however, almost all of the variability within the 18S gene was present within the most consistently amplified shorter segment. We found 11 distinct genotypes among US samples, eight in green turtles, two in loggerheads (including that of *E. carettae*), and one in both loggerheads and Kemp's ridleys (Table 2). Pairwise percent identity comparisons are shown in Table 3. Most examples of each genotype were represented by identical 18S and MT-*coI* sequences; however, some variability was present within the 18S of coccidia with the same MT-*coI* sequence. These exceptions included US6 and 9, which had 2 and 3 variants, respectively, of the 18S gene. We were able to sequence the larger 18S partial sequence from most examples. The US9 18S variants had between 1 and 6 nucleotide differences; a single nucleotide difference was found between variants of US6. The single base position difference in US6 variants was within the additional larger sequence that was inconsistently available for all genotypes, thus individual US6 variants do not appear in the tables. Similar to US genotypes and as noted by Chapman et al. (4), there are 4 variants of the Australian genotype 1, designated here (with GenBank accession numbers) as Aus1A (KT361639), Aus1B (MN450829), Aus1C (MN450830), and Aus1D (MN450831), with up to 4 nucleotide differences within the 18S and identical MT-*coI* sequence (MN450846). We also sequenced the MT-*coI* gene (MN450847) from the previously reported Australian genotype 2 (KT361640).

**Table 2:** Genotypes of coccidia found in stranded sea turtles in the US by host species (Cm = *Chelonia mydas*; Cc = *Caretta caretta*; Lk = *Lepidochelys kempii*), turtle straight carapace length (measured from nuchal notch to caudal suprapygal scute, median given if *n* > 4), locations of discovery, number of examples representing each genotype, and GenBank accession numbers for corresponding 18S ribosomal and mitochondrial cytochrome oxidase I gene (MT-coI) genes.

**Genotype**	**Host species**	**SCL median**	**SCL range**	**Location(s)**	**No. of turtle hosts**	**GeneBank (18S)**	**GenBank (MT-*coI*)**
1	Cm	28.6	17.4–50.4	US-Atl;HW	24	MN450815	MN450832
2	Cm	25.4	19.9–68.4	US-Atl	9	MN450816	MN450833
3	Cm	30.3	22.1–52.5	US-Atl,GoM	12	MN450817	MN450834
4	Cc	69.7	7.6–80.9	US-Atl	4	MN450818	MN450835
	Lk	-	34.0–52.4	US-Atl	2		
5^a^	Cc	68.2	-	US-Atl	1	MN450819	MN450836
6	Cm	27.3	23.7–49.7	US-Atl	5	MN450820	MN450837
7	Cm	49.2	-	US-Atl	1	MN450821	MN450838
8	Cm	49.5	-	US-GoM	1	MN450822	MN450839
9^b^	Cm	30.8	27.5–104.2	US-Atl;Aus	9^b^	MN450823-25	MN450840-42
10	Cm	32.1	-	US-GoM	1	MN450826	MN450843
11	Cc	72.7	-	US-GoM	1	MN450827	MN450844

Locations are provided as the Atlantic coast of the US (US-Atl), Gulf of Mexico coast of the US (US-GoM), Hawai'i (HW), and eastern Australia (Aus).

a Corresponds to Eimeria carettae.

b *The following are the number of individual cases for each of 3 variants of this genotype: 9A (n = 2); 9B (n = 4); 9C (n = 2)*.

**Table 3:**
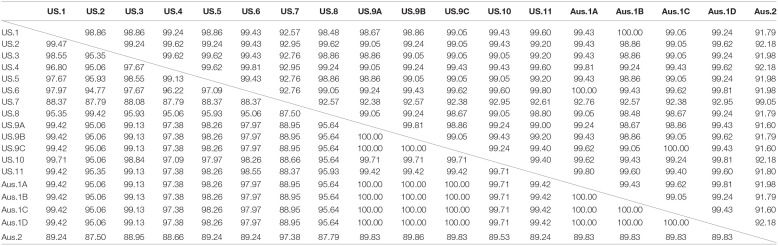
Percentage identity matrix of partial 18S ribosomal (above diagonal line) and mitochondrial cytochrome oxidase I (MT-*coI*, below diagonal line) genes of coccidia from green turtles (*Chelonia mydas*), loggerhead turtles (*Caretta caretta*), and Kemp's ridley turtles (*Lepidochelys kempii*) collected in the southeastern US and Australia (Aus).

*Genotype numbers reflect MT-coI sequences; letters are assigned to variants with variable 18S sequences, but identical MT-coI sequences. US5 corresponds to Eimeria carettae. All other genotypes are Caryospora-like based on morphological similarity to C. cheloniae*.

We found evidence of additional genetic diversity among cases from the southeastern US, but did not designate more genotypes because we were either unable to obtain unambiguous sequence, likely due to mixed infections, or we could not sequence either the 18S or MT-*coI* for undetermined reasons. These instances included loggerheads with US4. Although we were able to repeatedly obtain unambiguous MT-*coI* sequence from one loggerhead and both Kemp's ridleys with US4, three other loggerheads had ambiguous peaks at several positions suggesting the presence of other genotypes. In addition, there were two unique genotypes found in individual green turtles represented by 18S or MT-*coI* sequence only (MN450828 and MN450845, respectively).

#### Comparison of Genotypes With Life History, Geography, and Disease

The species, SCLs, and locations where infected turtles were found are given for each US genotype in Table 2. Most genotypes were only found in one host species; US4 was found in both loggerheads and Kemp's ridleys. All but two infected turtles were neritic phase juveniles within the size range typically found within habitat near where they stranded. Infected sea turtles of other life phases that we documented included a post-hatchling loggerhead (SCL = 7.6 cm) with US4 and an adult green turtle (SCL = 104.2 cm) with US9.

Coccidia from green turtles found prior to the Florida epizootic, including the first detected case in a free-ranging green turtle in 2007 and one of the initial stranded green turtles found in Florida in 2012, were genotypes US6 and 3, respectively. Of the 19 cases with sequence data from the 2014/15 Florida epizootic, all but one was US1. In contrast, of sequences from 35 green turtles found after the Florida epizootic, four were US1, seven were US2, eight were US3, four were US6, eight were US9, one each were US7, US8, and US10, and one was a mixed infection of US2 and US3 evidenced by clear double peaks at the expected locations in the chromatographs.

Coccidia infecting the stranded green turtle found in Hawai'i were also US1. As we were surprised to find identical genotypes in different ocean basins, we also sequenced the two ITS regions to further examine genetic similarity. Examples of US1 from the southeastern US and Hawai'i were identical across 18S, MT-*coI*, and both ribosomal spacer regions (MN450848).

In addition, the 18S and MT-*coI* of US9C genotype were identical to a genotype found in Australia, Aus1C. We were able to sequence most of the ITS2 and found 4/382 nucleotide differences between US9C (MN450849) and Aus1C (MN450850). Multiple attempts to obtain sequences for the ITS1 of these genotypes failed.

When genotypes were compared with clinical history and necropsy findings, some infections with US1 (*n* = 16), US2 (*n* = 4), US3 (*n* = 1), US9B (*n* = 1), US11 (*n* = 1), and a mixed infection of US2 and US3 (*n* = 1) were regarded as clinically significant. Clinical significance of the remainder was unclear. Coccidial enterocolitis was confirmed by histopathology in turtles parasitized by US1 (*n* = 16), 2 (*n* = 2), and 9B (*n* = 1) with the small intestine being the most affected in examples of US1 and 9B (Figures 2A,B). In three cases with US1 or US2, the most prominent gross lesion was ulcerative colitis covered by tenacious exudate (Figures 2C,D). Histopathological examination included the brain and other major organs of 14 turtles infected with US1 and one turtle with US9B; no coccidia were found in extra-intestinal tissues. All other genotypes were derived from coccidia found in feces of live turtles that survived, thus necropsy data were not available.

#### Phylogenetic Analysis

**Figure 5:**
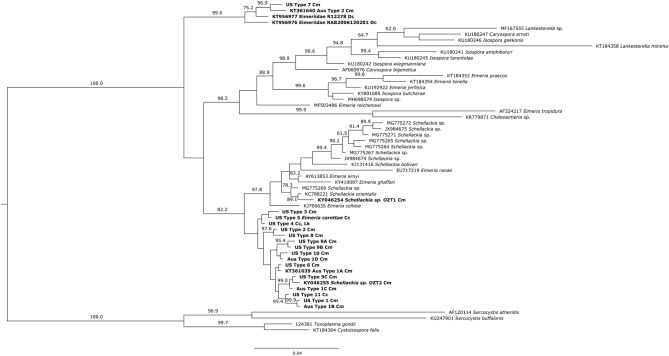
Bayesian inference analysis of partial 18S sequences from sea turtle coccidia and related taxa. Bold type indicates sequences obtained from sea turtle coccidia; host species is indicated by abbreviations following sequence name (Cm = *Chelonia mydas*; Cc = *Caretta caretta*; Dc = *Dermochelys coriacea*; Lk = *Lepidochelys kempii*). Numbers on branches indicate posterior probabilities as a percentage; values under 60% are not shown. Scale bar indicates the number of nucleotide substitutions per site.

**Figure 6:**
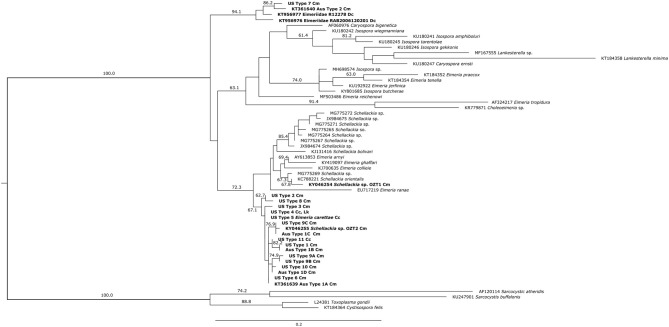
Maximum likelihood analysis of partial 18S sequences from sea turtle coccidia and related taxa. Bold type indicates sequences obtained from sea turtle coccidia; host species is indicated by abbreviations following sequence name (Cm = *Chelonia mydas*; Cc = *Caretta caretta*; Dc = *Dermochelys coriacea*; Lk = *Lepidochelys kempii*). Numbers on branches indicate bootstrap values as a percentage; values under 60% are not shown. Scale bar indicates the number of nucleotide substitutions per site.

The Bayesian Inference and Maximum Likelihood analyses using the 18S gene produced phylogenetic trees of similar topology (Figures 5, 6). The US and Australian sequences included in this study formed two clades. The first, containing all Australian genotype 1 variants and all US genotypes but one, was sister to another clade containing the *Schellackia* and several species of *Eimeria* infecting amphibians and reptiles. The second clade was comprised of US Type 7, Australian Type 2, and two Eimeriid coccidian sequences obtained from leatherback turtles (KT956976 and KT956977), and was basal to another containing species of *Isospora* and *Eimeria*. Notably, two sequences from apicomplexans infecting green turtles in Australia, obtained from Genbank, were split between the first, larger clade containing our sea turtle coccidia (KY046255) and the *Schellackia*/*Eimeria* clade (KY046254). Similar analysis of the MT-*coI* did not yield additional resolution (data not shown).

## Discussion

We describe the first epizootic of CLOs in free-ranging sea turtles in the northern hemisphere and report observations related to coccidial infections among stranded turtles in the US in recent years. Although we detected inter- and intraregional genetic variation among coccidia with oocysts that resemble previous descriptions of *C. cheloniae*, we found identical or highly similar genotypes in both Atlantic and Pacific green turtles based on genes known to exhibit interregional diversity in other species of Apicomplexa (18, 19, 26). Of particular concern with regard to wildlife health is that these genotypes were found during epizootics involving green turtles in the US and Australia.

The first report of *C. cheloniae* in maricultured turtles at a facility in the Cayman Islands predated molecular methods. Researchers relied on morphology for later comparisons and attributed the first epizootic involving wild green turtles in Australia to *C. cheloniae* based on the original description (9). Chapman et al. (4) provided the first genetic data on CLOs in Australian green turtles from a later epizootic, demonstrating genetic differences among organisms associated with the outbreak and noting problems with the current taxonomy. Our analyses are in agreement with previous studies, showing at least three distinct clades in the Eimeriidae that are all called *Caryospora*, in addition to the paraphyly of *Eimeria, Schellackia, Lankesterella*, and *Isospora*. Phylogenetic classification of coccidia based on sporulation patterns has proven problematic due to significant homoplasy in this characteristic. Gene sequence data have often been useful in determining which morphologic/life cycle characteristics of coccidians are phylogenetically informative. Using molecular data, organisms previously all labeled as *Isospora* based on sporulation patterns were recognized as *Isospora* and *Cystoisospora*, indicating that the presence or absence of Stieda bodies, the use of paratenic hosts, and avian or mammalian host specificity, are more phylogenetically informative than the number of sporocysts or sporozoites for these genera (27). Indeed, some of the *Cystoisospora* inconsistently undergo sporulation patterns that would cause them to be misidentified as *Caryospora* (28, 29). Revision of current taxonomy is expected, and the CLOs of sea turtles may well prove to be a novel genus; this will require sequence data of *C. simplex* to determine.

In the US, resource agencies, academic programs, and rehabilitation organizations have studied sea turtle strandings through clinical evaluation and necropsy in the decades following the original Caribbean epizootic. Although there have been parasitological descriptions of coccidia in loggerheads and leatherbacks (16, 30) and numerous studies of diseases affecting green turtles in the Northwest Atlantic and Central Pacific [e.g., (31, 32)], there were no reports of enteric coccidiosis associated with morbidity or mortality of wild sea turtles in these regions prior to 2012. Recent attention resulting from the 2014/15 US coccidiosis epizootic undoubtedly facilitated discovery of subsequent cases detected in our study; however, given the previous surveillance effort, we believe clinically significant enteric coccidiosis is a newly emergent disease of green turtles in the northern hemisphere.

Genetic characterization allowed us to demonstrate that the Florida epizootic was largely attributable to coccidia of a single genotype, US1, and that this genotype also was linked to the first case of enteric coccidiosis identified in Hawai'i. In addition, we found another coccidian genotype in two stranded green turtles in Florida that was highly similar to one characterized during an Australian epizootic in 2014. These findings show lack of variability across the three different genetic loci most commonly used to study intraspecific diversity in a variety of taxa. Limited genetic diversity among parasites and lack of geographical relation are indications of pathogen emergence and anthropogenic spread (33, 34). Although additional studies are prudent, our findings indicate a substantial probability that pathogenic coccidia of sea turtles have undergone recent dissemination among ocean basins.

Large-scale spread of coccidia could occur though transportation or migration of infected hosts or transport of oocysts. Although movement or comingling of infected sea turtles within ocean basins is possible, interoceanic movement is highly improbable. The separation of Atlantic and Indo-Pacific green turtle populations is ancient with minimal recent gene flow (35). Infected intermediate hosts also may spread coccidia. Low intraspecific variation is described in *Sarcocystis* spp. that use birds as intermediate hosts (36). It is unknown whether CLOs found in sea turtles have direct or indirect life cycles (4). Regardless, we are unaware of any candidate intermediate hosts that naturally move between the Pacific and Atlantic Oceans. There are numerous examples of recent range shifts by marine organisms associated with warming sea temperatures that could influence pathogen distribution; however, most of the shifts studied to date have been latitudinal within ocean basins (37).

Anthropogenic transport of oocysts or infected intermediate hosts is another possibility. The resilience of *Caryospora* oocysts have not been specifically studied, but coccidian oocysts in general are well-known for their stability in the environment and resistance to chemical disinfection (38). It is plausible that oocysts or infected intermediate hosts could be transported between distant locations in ballast water aboard ships. Ballast water is known to be a potential means of transport of marine organisms, including protozoa, algae, and invertebrates (39–41). Examination of seawater, ballast water, and potential intermediate hosts for CLOs would be valuable in exploring possible avenues of transmission and associated potential risks of human-mediated dissemination of coccidia.

As with locality, there was no apparent phylogenetic structuring of the coccidia by host species, which suggests a history of host switching rather than diversification through prolonged species coevolution. Detection of US4 in both loggerhead and Kemp's ridley turtles was somewhat unsurprising as these species have similar diets and are hosts for a number of the same metazoan parasites (42, 43). In addition, our phylogenetic analyses did not provide further insight into apparent differences in tissue distribution of coccidia, which is one of the most remarkable dissimilarities among reports from Australia and those from the northern hemisphere. Coccidia have been consistently described in extra-intestinal locations, including the brain, thyroid gland, and kidneys in Australian epizootics, accompanied by neurological signs, whereas organisms have only been observed thus far in the mucosa of the digestive tract in US cases. Histopathological findings from green turtles found in the US with confirmed CLO infection included examination of the brain and other organs from a total of 38 green turtles, including 14 with US1, one with US9B, and 23 from the 2014/15 epizootic where genotype was not identified, but was most likely US1. Because many of the US genotypes were found in live turtles that survived, including US9C with its high similarity to Aus1C, additional study of tissue distribution is required.

We do not yet have enough information to compare differences in pathogenicity among the coccidian genotypes. Some of the genotypes, especially US1 and those found in Australia, clearly cause significant disease in some sea turtles, but others were detected without clinically apparent effect or possible masking of any signs by concurrent health problems, including coccidia found in loggerheads and Kemp's ridleys. Turtles without evident clinical signs that are shedding oocytes may have had enteric lesions of limited distribution or that were uncomplicated by secondary bacterial infection. Subclinical shedding may pose a risk to other sea turtles, especially in captive situations, thus regular fecal examinations using flotation methods are a sensible component of initial clinical evaluation and subsequent assessments.

Stranded sea turtles with coccidia have occurred in Australia during spring, leading to a hypothesis that warming water temperatures may play a role in development of clinical disease (4). However, at least in the southeastern US, sea turtle strandings peak in the spring regardless of cause, so it is unclear whether seasonality of coccidial infections exists in this region.

Previous reports of CLO infections in sea turtles include all life stages from hatchlings to adults (4, 8, 9). In the first reported Australia epizootic, most affected turtles were immature (9), whereas adult turtles were thought more susceptible during a subsequent large outbreak (4). The median SCL of green turtles affected during the Florida epizootic was lower than concurrent strandings of the same species that did not meet our case definition, suggesting that smaller juveniles were disproportionately affected. However, we did not sufficiently screen turtles of other sizes during the epizootic to explore more confidently the relationship between size and CLO infection. Given the inherent biases related to study of stranded sea turtles, future studies examining parasite prevalence in free-ranging sea turtles during the course of in-water sea turtle research are needed in order to better understand prevalence among size classes.

Enteric coccidiosis resulting from CLOs is one of very few known infectious causes of episodic mass stranding and mortality events involving sea turtles. Although study of epizootics has characterized the pathology of CLO infections and the demography of affected turtles, many aspects of these parasites remain poorly understood, including their life cycle and dynamics of infection in free-ranging sea turtle populations. Multi-institutional, international collaboration in the current study allowed us to examine CLO infections for the first time over a large spatial scale and to undertake intensive screening and sampling of stranded turtles to gain a more complete appreciation of the genetic diversity of these parasites and their global distribution. Moreover, the discovery of genetically identical organisms in different ocean basins raises significant concerns regarding the potential for human-mediated spread of these sea turtle pathogens.

## Data Availability Statement

The raw data supporting the conclusions of this manuscript will be made available by the authors, without undue reservation, to any qualified researcher.

## Ethics Statement

The animal study was reviewed and approved by University of Florida IACUC.

## Author Contributions

All authors contributed to data and sample collection and review and preparation of the manuscript. In addition, data analyses were conducted by BS, PC, HS-W, TW, JD, AF, MW, and JW.

### Conflict of Interest

MR is employed by Miami Seaquarium and LS is employed by SeaWorld Orlando. The remaining authors declare that the research was conducted in the absence of any commercial or financial relationships that could be construed as a potential conflict of interest.
